# Reply to comments on: “Obesity associated with a novel mitochondrial tRNA^Cys^ 5802A>G mutation in a Chinese family”

**DOI:** 10.1042/BSR20200131

**Published:** 2020-02-11

**Authors:** Jinling Wang, Ningning Zhao, Xiaoting Mao, Feilong Meng, Ke Huang, Guanping Dong, Yanchun Ji, JunFen Fu

**Affiliations:** 1 Department of Endocrinology, The Children’s Hospital, Zhejiang University School of Medicine, National Clinical Research Center For Child Health, Hangzhou, China; 2 Division of Medical Genetics and Genomics, The Children’s Hospital, Zhejiang University School of Medicine, National Clinical Research Center For Child Health, Hangzhou, Zhejiang 310058, China; 3 Institute of Genetics, Zhejiang University, Hangzhou, Zhejiang 310058, China

**Keywords:** mitochondrial tRNA, mutation, obesity

Dear Editor,

We thank Dr. Finsterer for the inquiry regarding the report about “Responsibility of the variant m.5802A>G in tRNA(Cys) for mitochondrial obesity remains unproven” [1]. Through mitochondrial gene sequencing, bioinformatics analysis and phylogenetic analysis, we believe that this mitochondrial tRNA mutation site may be a risk factor related to obesity. In response to his questions, we make the following explanation:

The first is the heteroplasmy rate of the m.5208A>G variant. We have mentioned that the tRNA^Cys^ 5802A>G mutation site is homozygous in the article. This mutation is similar as homoplasmic tRNA^Thr^ 15927G>A mutation with coronary artery disease [2], not like heterplasmic tRNA^Ile^ 3243A>G with mitochondrial encephalopathy lactic acidosis stroke-like episodes (MELAS) [3]. Their pathogenic ways are different and cannot be compared. Therefore, it is one-sided to analyze them from heterogeneity.

Another is the pathogenicity of m.5802A>G mutation. We follow the principle that structure determines function. We analyzed the possible pathogenicity from the structure, position and molecular dynamics of this site, which indicated that this site is located in the highly conserved region of tRNA, and after mutation, it can change the structure and molecular dynamics of tRNA, which may affect the function of tRNA. Next, we will construct the lymphoblastoid cell lines, establish a model of trans-mitochondrial cybrid cell lines, and analyze the biochemical function such as in aminoacylation and steady-state levels of tRNA, mtDNA-encoded polypeptides, respiratory rates, membrane potential and the production of reactive oxygen species.

Mitochondrial DNA mutations are tissue-specific. Not all mutations show symptoms in the same tissues. For example, mitochondrial 1555A>G mutation mostly appears in deafness [4], mitochondrial 11778G>A mutation only appear in Leber’s hereditary optic neuropathy (LHON) [5] etc. Obesity is the only characteristic of the patient. Cerebrospinal fluid (CSF) examination is invasive. This examination is not recommended if there is no relevant physical sign. Moreover, the tRNA^Cys^ 5802A>G is homozygous and tissue-specific, which is not related to lactic acidosis as the Dr Finsterer suggested.

Therefore, as described in our article, a novel mitochondrial tRNA 5802A>G mutation was dected in a Chinese obese population. Through the analysis of structure and function prediction, it is considered that this site may be a possible inherited risk factor associated with obesity. However, the lower penetrance of obesity in this family suggested that other factors such as the nuclear modifier gene(s) or environmental factor(s) may play a role in the phenotypic manifestation of this mutation in these Chinese families. Further studies will be needed to definitively assess the relationship between mitochondrial dysfunction and the onset of obesity *in vivo*. Further research is needed to clearly evaluate the relationship between mitochondrial dysfunction and obesity *in vivo*.
